# Progression of Luminal Breast Tumors Is Promoted by Ménage à Trois between the Inflammatory Cytokine TNF**α** and the Hormonal and Growth-Supporting Arms of the Tumor Microenvironment

**DOI:** 10.1155/2013/720536

**Published:** 2013-12-04

**Authors:** Polina Weitzenfeld, Nurit Meron, Tal Leibovich-Rivkin, Tsipi Meshel, Adit Ben-Baruch

**Affiliations:** Ela Kodesz Institute for Research on Cancer Development and Prevention, Department of Cell Research and Immunology, George S. Wise Faculty of Life Sciences, Tel Aviv University, 69978 Tel Aviv, Israel

## Abstract

Breast cancer progression is strongly linked to inflammatory processes, aggravating disease course. The impacts of the inflammatory cytokine TNF**α** on breast malignancy are not fully substantiated, and they may be affected by cooperativity between TNF**α** and other protumoral mediators. Here, we show that together with representatives of other important arms of the tumor microenvironment, estrogen (hormonal) and EGF (growth-supporting), TNF**α** potently induced metastasis-related properties and functions in luminal breast tumor cells, representing the most common type of breast cancer. Jointly, TNF**α** + Estrogen + EGF had a stronger effect on breast cancer cells than each element alone, leading to the following: (1) extensive cell spreading and formation of FAK/paxillin-enriched cellular protrusions; (2) elevated proportion of tumor cells coexpressing high levels of CD44 and **β**1 and VLA6; (3) EMT and cell migration; (4) resistance to chemotherapy; (5) release of protumoral factors (CXCL8, CCL2, MMPs). Importantly, the tumor cells used in this study are known to be nonmetastatic under all conditions; nevertheless, they have acquired high metastasizing abilities *in vivo* in mice, following a brief stimulation by TNF**α** + Estrogen + EGF. These dramatic findings indicate that TNF**α** can turn into a strong prometastatic factor, suggesting a paradigm shift in which clinically approved inhibitors of TNF**α** would be applied in breast cancer therapy.

## 1. Introduction

The majority of breast cancer patients are diagnosed with luminal tumors that are characterized by the expression of estrogen receptors (ER) and progesterone receptors (PR) and the absence or only weak amplification of HER2 (this latter parameter depends on the subclass, whether luminal A or luminal B) [1, 2]. Although ER-expressing and PR-expressing patients typically experience a favorable outcome and a relatively good prognosis, eventually many of them become unresponsive to endocrine therapies and develop metastases at remote organs [1–3]. To date, the mechanisms that contribute to tumor progression and more importantly to metastasis formation in these patients are poorly understood.

Tumor cell dissemination to remote organs is a multifactorial process that is linked to upregulation of extracellular matrix (ECM) and adhesion receptors, to increased spreading and migration, and to epithelial-to-mesenchymal transition (EMT) [4–10]. Moreover, strong induction of metastatic traits is endowed on the tumor cells by elements of the tumor microenvironment that promote many different metastasis-related functions including tumor cell spreading and EMT [11–13].

The tumor milieu is an extremely complex and dynamic contexture comprised of many cell types, ECM components, and secreted factors. Recently, intensive research indicates that there is an intimate link between inflammation and cancer, where inflammatory cells and cytokines promote processes of tumor growth and progression. In this respect, much emphasis has been attributed to the inflammatory cytokine tumor necrosis factor *α* (TNF*α*). TNF*α* was shown to induce antitumor effects when administered in high concentrations directly into tumors. Thus, TNF*α* was considered for quite some time as a potential therapeutic modality, whose introduction to patients would limit disease course. However, recent investigations challenged this view and indicated that chronic and consistent presence of TNF*α* in tumors leads to procancerous consequences in many malignant diseases [14–17].

Specifically in breast cancer, studies in animal model systems have shown that TNF*α* exerted causative procancerous activities through a diverse set of mechanisms [18–21]. Along these lines, we and others have shown that TNF*α* was highly expressed in breast tumors [22–25], that the incidence of TNF*α* expression was significantly increased in advanced stages of breast cancer (detected in approximately 90% of the patients with recurrent disease) [22], and that TNF*α* induced EMT and invasion of breast tumor cells [22, 26, 27]. Moreover, by virtue of its inflammatory actions as inducer of inflammatory chemokines, TNF*α* indirectly led to high presence of protumoral leukocyte subpopulations in tumors [28].

The opposing roles of TNF*α* in cancer may be due to interactions that the cytokine has with other procancerous elements that reside at the tumor milieu. In luminal breast tumors, such interactions could be taking place mainly with two arms of the tumor microenvironment: hormones that are key regulators of the malignant process and growth-supporting factors that promote tumor cell proliferation. Indeed, the hormone estrogen is a key player in luminal breast tumors, where it enhances the proliferation of breast tumor cells, induces EMT, and consequently increases the migratory and invasive abilities of these cells [29–32]. Although the lack of ER is usually associated with worse prognosis [32, 33], the hormone by itself has definite potent tumor-promoting functions and thus is a major therapeutic target in breast cancer treatment. In parallel, growth-supporting factors like epidermal growth factor (EGF) are of large relevance. Luminal breast cancer cells usually do not exhibit amplification of the EGF-signaling HER2 receptor or show only low over-expression of this receptor; nevertheless, they bind EGF and respond to its tumor-promoting stimuli [34–37]. EGF enhances tumor cell proliferation, migration, invasion, and EMT [36, 38–40], and thus it should be taken into account when we consider joint activities of microenvironmental factors on breast cancer metastasis.

In view of the multi-factorial nature of the tumor microenvironment, in this study we determined the combined impact of the three arms—inflammatory (TNF*α*) + hormonal (estrogen) + growth-supporting (EGF)—on malignancy-promoting characteristics and functions of luminal breast tumor cells. This “combined stimulation” by TNF*α* + Estrogen + EGF provides a more relevant representation of the multifaceted nature of the tumor microenvironment in luminal breast tumors than the reductionist approach of testing the activity of each element alone. The “combined stimulation” approach is supported by published findings demonstrating coregulatory intracellular interactions existing between TNF*α*-, estrogen-, and/or EGF-mediated pathways in breast cancer and in other malignancies [34, 41, 42]. Accordingly, in this study we determined the impact of the TNF*α* + Estrogen + EGF stimulus and compared it to the effect of each factor on its own. Using the joint powers of the TNF*α* + Estrogen + EGF stimulation, we found that MCF-7 luminal breast tumor cells have acquired very high metastasis-related functions. Already at the initial phases of the study we found that the combined stimulation had a much higher influence than TNF*α* alone, estrogen alone, or EGF alone on the tumor-promoting aspects that were studied. Therefore, in more advanced stages of the research we focused on the effects of the joint stimulation by TNF*α* + Estrogen + EGF on functional tumor-promoting readouts, including tumor growth and metastasis formation.

Overall, our findings indicate that TNF*α* induces many metastasis-related functions in luminal breast tumor cells and that its activities are largely amplified by cooperativity with estrogen and EGF. The TNF*α* + Estrogen + EGF stimulation has endowed the cancer cells with high spreading and EMT characteristics and with tumor- and metastasis-promoting functions. Moreover, although TNF*α* was cytotoxic to some of the tumor cells, its cooperativity with estrogen and EGF has led to selection of tumors cells that have gained high metastasizing abilities *in vivo*, in an animal model system.

These observations suggest that a paradigm shift is required in the treatment of luminal breast cancer patients, in which therapies against TNF*α* should be introduced to the clinical regimen rather than the use of TNF*α* as a cytotoxic agent. Inhibitors of TNF*α* are already in clinical use for other indications (autoimmune diseases), and our findings suggest that they should be combined with antihormonal approaches and modalities targeting the EGF-HER2 pathway. We propose that such integrative therapies targeting multiple tumor-promoting factors may achieve a high therapeutic impact in luminal breast cancer patients.

## 2. Materials and Methods

### 2.1. Cell Cultures

MCF-7 cells are luminal breast tumor cells that express high levels of ER and PR and show low levels of expression of HER2 and of EGF receptors (EGFR) [43–45]. These cells were found to provide the unique setup of luminal breast tumor cells which is required for this study by (1) responding to TNF*α* [22, 46, 47]; (2) expressing estrogen receptor *α* (ER*α*) and responding to estrogen [34, 48, 49]; (3) responding to EGF despite relatively low expression of HER2 and EGFR [34–37]. The cells were kindly provided by Professor Kaye (Weizmann Institute of Science, Rehovot, Israel). In line with published MCF-7 characteristics [43–45], the cells were authenticated on the basis of expression and activity of ER*α*; *in vitro* estrogen responsiveness; tumor formation requiring estrogen and matrigel; and low expression of HER2. The cells were maintained in growth media containing DMEM supplemented by 10% fetal calf serum (FCS), 2 mM L-glutamine, 100 Units/mL penicillin, 100 *μ*g/mL streptomycin, and 250 ng/mL amphotericin (all from Biological Industries, Beit Haemek, Israel).

### 2.2. Cell Stimulation

MCF-7 cells were plated over-night in complete media, washed in PBS, and stimulated for three days with TNF*α*, estrogen, and/or EGF. The concentrations of the three stimulants were selected based on extensive titration and kinetics analyses (data not shown), and they agree with the conventional dose range used in other research systems: TNF*α* at 50 ng/mL (cat. no. 300-01A; PeproTech, Rocky Hill, NJ, USA), estrogen at 10^−8 ^M (cat. no. E8875; Sigma, Saint Louis, MO, USA) and EGF at 30 ng/mL (cat. no. 236-EG; R&D systems, Minneapolis, MN, USA). In all procedures, control non-stimulated cells were grown in the presence of the diluents of the above stimulators. Stimulation was performed in phenol red-free and serum-free DMEM. Media, including the stimulators, were changed daily.

When indicated, the pharmacological inhibitor of Src, PP2 (cat. no. 529573; Calbiochem, EMD Millipore, San Diego, CA, USA) was used in a conventional concentration of 2.5–5 *μ*M. The inhibitor was added to cell cultures simultaneously with the stimulation of the cells by TNF*α* + Estrogen + EGF or to control non-stimulated cells and was present in culture throughout the duration of stimulation (three days). Control cells were treated with the solubilizer of the drug at similar dilutions (DMSO; Sigma).

### 2.3. Confocal Microscopy Analyses

Stimulated and non-stimulated MCF-7 cells were fixed with 8% paraformaldehyde (PFA; cat. no. 1.04005; Merck KGaA, Darmstadt, Germany), permeabilized by 0.2% Triton (cat. no. X-100; Sigma), and blocked with 2% BSA (cat. no. 0332-TAM; Amresco, Solon, OH, USA). Nuclei were visualized by DAPI (4′,6-diamidino-2-phenylindole; cat. no. 9564; Sigma) and actin fibers by FITC-conjugated phalloidin (cat. no. P-5282; Sigma). The following antibodies (Abs) were used: rabbit IgG against focal adhesion kinase (FAK; cat. no. sc-558; Santa Cruz biotechnology, Santa Cruz, CA, USA) and mouse IgG1 against paxillin (cat. no. 624001; Biolegend, San Diego, CA, USA). Then, the cells were incubated with the secondary Abs: Dylight-549-conjugated against rabbit IgG (cat. no. 111-505-144; Jackson Immunoresearch Laboratories, West Grove, PA, USA) or Alexa-647-conjugated against mouse IgG (cat. no. 115-606-146; Jackson Immunoresearch Laboratories). Baseline staining was obtained by nonrelevant isotype matched controls. Coverslips were mounted using fluorescent mounting medium (cat. no. E18-18; Golden Bridge International, Mukilteo, WA, USA) and read by Zeiss LSM 510-META confocal microscope (Carl Zeiss, Jena, Germany) at ×63 magnification.

### 2.4. Flow Cytometry

Expression levels of cell surface molecules were determined by flow cytometry (FACS) in stimulated and non-stimulated MCF-7 cells, using a Becton Dickinson FACSort (Mountain View, CA, USA). The following Abs were used: PE-conjugated mouse IgG1 against integrin *β*1 (CD29; cat. no. 303004; Biolegend), Alexa 488 conjugated-Rat IgG2a against integrin *α*6 (CD49f; cat. no. 313607; Biolegend), Alexa 488-conjugated Rat IgG2b against CD44 (cat. no. 103015; Biolegend) and mouse IgG1 against E-cadherin (Figure 7—cat. no. sc-21791; Santa Cruz biotechnology; Figure S2—cat. no. 324101; Biolegend). The Abs against E-cadherin were followed by FITC-conjugated Abs against mouse IgG (cat. no. 115-095-003; Jackson Immunoresearch Laboratories). Baseline staining was obtained by nonrelevant isotype matched controls. Staining patterns were determined using the win MDI software.

### 2.5. Quantitative Real-Time Polymerase Chain Reaction

Total RNA was isolated from stimulated and non-stimulated MCF-7 cells using the EZ-RNA kit (cat. no. 20-400-100; Biological Industries). RNA samples were used for generation of first-strand complementary DNA synthesis using the M-MLV reverse transcriptase (cat. no. AM2044; Ambion, Austin, TX, USA). Quantification of cDNA targets by quantitative real-time polymerase chain reaction (qPCR) was performed on Rotor Gene 6000 (Corbett Life Science, Concorde, NSW, Australia), using Rotor Gene 6000 series software. Transcripts were detected using Absolute Blue qPCR SYBR Green ROX mix (cat. no. AB-4163/A; Thermo Fisher Scientific, Waltham, MA, USA) according to the manufacturer's instructions. In each reaction, two pairs of specific primers were used, designed for different exons. The sequences of the primers as follows: for Zeb1-forward 5′-TGCAGCTGACTGTGAAGGTGT-3′, reverse 5′-CTTGCCCTTCCTTTCTGTCATC-3′; for Snail-forward 5′-CTAATCCAGAGTTTACCTTCCAGCA-3′, reverse 5′-AGTCCCAGATGAGCATTGGC-3′; for Slug-forward 5′-CCTGGTCAAGAAGCATTTCAA-3′, reverse 5′-CAGGCATGGAGTAACTCTCA-3′; for the normalizing gene rS9-forward 5′-TTACATCCTGGGCCTGAAGAT-3′ and reverse 5′-GGGATGTTCACCACCTGCTT-3′. PCR amplification of the genes rS9 and Slug was performed over 40 cycles (95°C for 15 sec, 59°C for 20 sec, 72°C for 15 sec), while amplification of Zeb1 was performed over 40 cycles (95°C for 15 sec, 59°C for 20 sec, 80°C for 15 sec), and of Snail over 45 cycles (95°C for 15 sec, 59°C for 20 sec, 84.5°C for 15 sec). Dissociation curves for each primer set indicated a single product, and no-template controls were negative after 40/45 cycles. Quantification was performed by standard curves, on the linear range of quantification.

### 2.6. Cell Viability

Stimulated and non-stimulated MCF-7 cells were recultured in 96-well plates in growth medium containing the stimulants. After 8 hr, media were removed and the cells were exposed to combined stimulation by TNF*α* + Estrogen + EGF, in the absence or presence of 1 *μ*M doxorubicin (Teva Pharmaceutical, Netanya, Israel; kindly provided by Professor Peer, Tel Aviv University). The concentration of doxorubicin was selected following titration analyses (data not shown). After additional three days, media were removed, cells were washed, and XTT reagent (cat. no. 20-300-1000; Biological Industries) was added to the wells according to the manufacturer's instructions for 2 hr. Absorbance was measured at 450 nm and 630 nm. For each group (non-stimulated and stimulated cells) the percentage of cell survival was calculated compared to cells that were not exposed to doxorubicin. In other cases, cell viability was determined by trypan blue exclusion (cat. no. 03-102-1B; Biological industries), in two replicates. Viable cells were counted using a hemocytometer, and total cell number was calculated.

### 2.7. ELISA Assays

Stimulated and non-stimulated MCF-7 cells were grown as described above. Conditioned medium (CM) was removed from the last 24 hr of cultures, and CXCL8 and CCL2 levels were determined by ELISA using standard curves with rhCXCL8 or rhCCL2 (cat. no. 200-08, 300-04, resp.; PeproTech), at the linear range of absorbance. The following Abs were used (all from PeproTech): For CXCL8: coating polyclonal Abs (cat. no. 500-P28), detecting biotinylated rabbit polyclonal Abs (cat. no. 500-P28Bt); For CCL2: coating monoclonal Abs (cat. no. 500-M71), detecting biotinylated rabbit polyclonal Abs (cat. no. 500-P34Bt). After the addition of streptavidin-horseradish peroxidase (cat. no. 016-030-084; Jackson Immunoresearch Laboratories), the substrate TMB/E solution (cat. no. ES001; Millipore, Temecula, CA, USA) was added. The reaction was stopped by the addition of 0.18 M H_2_SO_4_ and was measured at 450 nm. In parallel, cells were removed by trypsinization and counted by trypan blue exclusion (see above), and the results were normalized to cell numbers.

### 2.8. Gelatin Substrate Zymography

MCF-7 cells were plated in 24-well plates in growth medium. Following overnight incubation, the growth medium was removed and cells were stimulated for three days with TNF*α* + Estrogen + EGF as indicated above. CM of the last 24 hr were collected and separated on 7.5% SDS-polyacrylamide gels containing 0.1% gelatin substrate. After electrophoresis, gels were washed three times in 50 mM Tris/HCl pH 7.5, containing 2.5% Triton X-100. The gels were then washed three times in 50 mM Tris/HCl pH 7.4 buffer, followed by incubation in buffer containing 50 mM Tris/HCl pH 7.4, 0.02% NaN_3_, and 10 mM CaCl_2_ for 48 hr at 37°C. Following three washes in double distilled H_2_O, the gels were stained with 0.1% coomassie blue and distained in 20% methanol and 10% glacial acetic acid until clear bands of protein degradation were visualized. In parallel, cells that were removed by trypsinization were counted by trypan blue exclusion (see above). The obtained bands were subjected to densitometry performed using Scion image software, and their density was normalized to cell number.

### 2.9. Assays of Tumor-Spheroids

6-well plates were incubated overnight on a rocker with 1.2% Poly(2-hydroxyethyl methacrylate) (cat. no. P3932; Sigma) in methanol. MCF-7 cells were plated in phenol-red free DMEM/F12 medium supplemented with 2 mM L-glutamine, 100 Units/mL penicillin, 100 *μ*g/mL streptomycin, 250 ng/mL amphotericin (all from Biological Industries), 0.4% BSA (cat. no. 0332-TAM; Amresco), B-27 serum-free supplement (cat. no. 17504; Gibco, Life Technologies, Grand Island, NY, USA), 20 ng/mL basic FGF (cat. no. 100-18B; Peprotech), 20 ng/mL EGF (cat. no. 236-EG; R&D systems), and 5 *μ*g/mL insulin (cat. no. I9278; Sigma). After three days, tumor-spheroids were formed, and cells were stimulated with TNF*α* + Estrogen + EGF in the above-indicated concentrations or with the diluents of the above stimulators, for additional 24–96 hr. Cells were photographed daily using a light microscope at ×10 magnification.

For flow cytometry analyses that followed tumor-spheroid formation, cells were passed through a 40 *μ*m nylon mesh cell strainer, in order to separate single cells from tumor-spheroids. Tumor-spheroids were later dissociated by trypsinization and the cells that were obtained by this procedure were compared to single cells that migrated out of tumor-spheroids formed by stimulated cells. Cell viability of all groups was determined by trypan blue exclusion (see above) and cells were stained using Abs against E-cadherin (see above).

### 2.10. Tumor Growth and Metastasis

MCF-7 cells were infected to stably express mCherry (by pQCXI-mCherry retroviral vector). The cells were either non-stimulated or stimulated by TNF*α* + Estrogen + EGF at the above-mentioned concentrations for three days. Then, the cells were washed and 4 × 10^6^ cells/mouse were inoculated to the mammary fat pad of female athymic nude mice (Harlan Laboratories, Jerusalem, Israel). Prior to injection to mice, the cells were mixed 1 : 1 with matrigel (cat. no. 356234; BD Biosciences, Franklin Lakes, NJ, USA). One week prior to tumor cell inoculation, all mice were implanted subcutaneously with slow-release estrogen pellets (1.7 mg/pellet, 60 days release, cat. no. SE-121; Innovative Research of America, Sarasota, FL, USA) which are essential for the growth of MCF-7 cells in mice.

The CRi Maestro noninvasive intravital imaging system was used to monitor intact mice, at four different time points along the time course of up to 37 days (depending on the experiment). The Maestro device has provided two readouts: (1) size of primary tumors along the growth process of tumors in the intact mice; (2) absence or presence of metastases in the intact mice. When the experiments were terminated, organs were excised and metastasis formation was compared to the readouts obtained by the Maestro device. This analysis has indicated that in intact mice, the Maestro device detected macro-metastases in a reliable manner, but could not detect micrometastases that may have been formed. Accordingly, the data retrieved by the Maestro device at the different time points in intact mice actually provided information on the formation of macrometastases in different organs.

The regulations of Tel Aviv University Animal Care Committee did not allow continuation of the experiments to the stage of survival analysis. All procedures involving experimental animals were performed in compliance with local animal welfare laws, guidelines, and policies.

### 2.11. Statistical Analyses

Statistical analyses of *in vitro* experiments were done using Student's *t*-tests. Values of *P* < 0.05 were considered statistically significant, and all *in vitro* data were presented as mean ± standard deviation (SD). In the *in vivo* studies of primary tumors, data are presented as mean ± standard error of mean (SEM), and statistical analyses of tumor sizes were done using Student's *t*-tests, where values of *P* < 0.05 were considered statistically significant.

## 3. Results

### 3.1. Combined Stimulation by TNF*α* + Estrogen + EGF Amplifies Tumor Cell Remodeling and Leads to Increased Cell Spreading and High Expression of Metastasis-Related Adhesion Molecules

TNF*α*, estrogen, and EGF were each shown to have the potential to promote metastasis-related properties in breast tumor cells, as described above; however, different research systems were used for the study of each of these factors. In our study, we have compared side by side the ability of TNF*α*, estrogen, and/or EGF to affect spreading and EMT properties, using the MCF-7 luminal breast tumor cells. These cells express receptors for all the three above-mentioned factors (references provided above), and represent a nonadvanced stage of breast malignancy that can be pushed forward towards a more aggressive/invasive phenotype in terms of acquisition of EMT properties [22, 26, 27].

**Figure 1:**
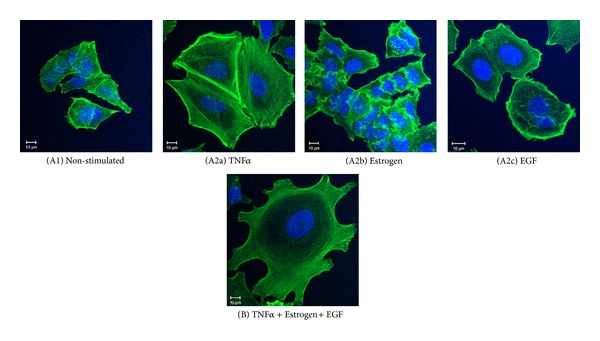
Combined stimulation by TNF*α* + Estrogen + EGF induces extensive morphological changes and spreading in breast tumor cells. Breast tumor cells were either (A1) not stimulated (cells grown in the presence of diluents) or stimulated by (A2a) TNF*α* (50 ng/mL), (A2b) estrogen (10^−8 ^M), (A2c) EGF (30 ng/mL), or (B) TNF*α* + Estrogen + EGF (concentrations as above) for three days. The stimulatory conditions were selected following titration and kinetics analyses (data not shown). Actin filaments were detected by phalloidin staining (green) and cell nuclei by DAPI staining (blue). The cells were analyzed by confocal microscopy. In all panels, the results are from a representative experiment of *n* ≥ 3.

First, we determined the effects of TNF*α*, estrogen, EGF, or all three factors together on tumor cell morphology, spreading and expression of adhesion molecules which promote tumor cell invasion and metastasis [50–53]. Stimulating the tumor cells for three days by TNF*α* has induced the formation of actin-rich cellular protrusions, accompanied by definite concentration of actin fibers at the cell cortex (Figure 1(A2a)). In contrast, estrogen alone had no effect on tumor cell morphology (Figure 1(A2b)), and EGF induced cell spreading but to lower extent than TNF*α* (Figure 1(A2c)). However, the most robust change in cell morphology, exemplified by extensive spreading and reorganization of stress fibers, was noted when estrogen and EGF were added to TNF*α* (Figure 1(B)). The cells that were exposed to the combined stimulation by TNF*α* + Estrogen + EGF have formed definite and large cellular protrusions, with actin stress fibers clearly apparent, which were minimally visible previously in the control non-stimulated cells (Figure 1(B) versus Figure 1(A1)). Additional analyses indicated also that the triple stimulation of TNF*α* + Estrogen + EGF was more effective in inducing spreading and cell remodeling than dual stimulations by Estrogen +TNF*α* or Estrogen + EGF (Figure S1 in Supplementary Material available online at http://dx.doi.org/10.1155/2013/720536) (the dual stimulations focused on combinations including estrogen because it is the most relevant factor to the luminal tumor cells we were using, characterized by ER expression). Together, these results provide evidence to strong impact of the combined stimulation by TNF*α* + Estrogen + EGF over other combinations, indicating that the joint activity of all three arms of the tumor microenvironment together is advantageous in inducing spreading and adhesion-related functions in luminal breast tumor cells.

**Figure 2:**
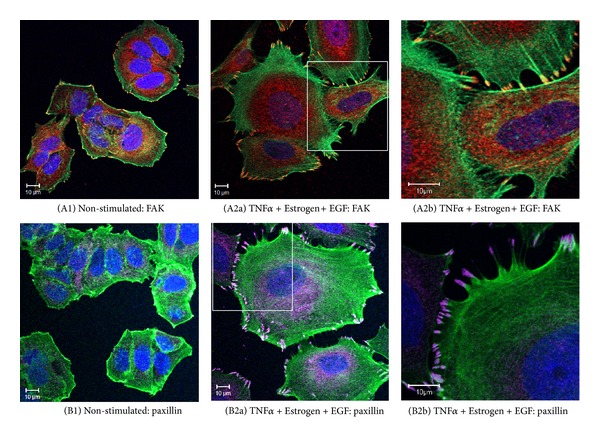
Combined stimulation of breast tumor cells by TNF*α* + Estrogen + EGF induces the localization of FAK and paxillin in cell protrusions and formation of intertumor connecting tubes. Breast tumor cells were stimulated by TNF*α* + Estrogen + EGF (concentrations as in Figure 1) for three days. Non-stimulated: cells grown with the diluents of the above factors. (A) The expression of FAK. (A1) Non-stimulated cells. ((A2a), (A2b)) Cells stimulated by TNF*α* + Estrogen + EGF, where part (A2b) demonstrates the formation of tubes connecting between different tumor cells. FAK expression was detected by specific Abs (red), actin filaments by phalloidin staining (green), and cell nuclei by DAPI staining (blue). In all panels, the results are from a representative experiment of *n* ≥ 3. (B) The expression of paxillin. (B1) Non-stimulated cells. ((B2a), (B2b)) Cells stimulated by TNF*α* + Estrogen + EGF, where part (B2b) demonstrates the formation of tubes connecting between different tumor cells. Paxillin expression was detected by specific Abs (purple), actin filaments by phalloidin staining (green), and cell nuclei by DAPI staining (blue). In all panels, the results are from a representative experiment of *n* ≥ 3.

**Figure 3:**
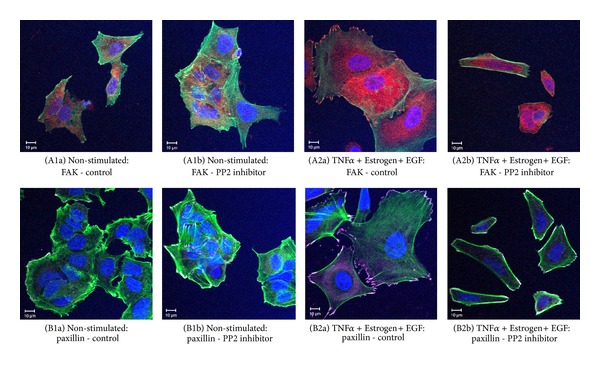
Cell-remodeling of breast tumor cells, induced by TNF*α* + Estrogen + EGF, depends on Src-induced mechanisms. Breast tumor cells were stimulated by TNF*α* + Estrogen + EGF (concentrations as in Figure 1) for three days. Non-stimulated: cells grown with the diluents of the above factors. The cells were either not exposed or exposed to the Src inhibitor PP2 (used at the range of 2.5–5 *μ*M). (A) The expression of FAK, determined in the absence ((A1a), (A2a)) or in the presence ((A1b), (A2b)) of PP2 (2.5 *μ*M in this specific experiment) in non-stimulated cells ((A1a), (A1b)) or in cells stimulated by TNF*α* + Estrogen + EGF ((A2a), (A2b)). FAK expression was detected by specific Abs (red), actin filaments by phalloidin staining (green), and cell nuclei by DAPI staining (blue). In all panels, the results are from a representative experiment of *n* ≥ 3. (B) The expression of paxillin, determined in the absence ((B1a), (B2a)) or in the presence ((B1b), (B2b)) of PP2 (2.5 *μ*M in this specific experiment) in non-stimulated cells ((B1a), (B1b)) or in cells stimulated by TNF*α* + Estrogen + EGF ((B2a), (B2b)). Paxillin expression was detected by specific Abs (purple), actin filaments by phalloidin (green), and cell nuclei by DAPI staining (blue). In all panels, the results are from a representative experiment of *n* = 3.

Additional analyses indicated that the morphological changes induced by TNF*α* + Estrogen + EGF in the tumor cells were accompanied by redistribution of focal adhesion kinase (FAK) and paxillin, two key regulators of cell adhesion and spreading [50–53] (Figure 2). Moreover, we noticed that the cancer cells that were exposed to the combined stimulation by TNF*α* + Estrogen + EGF have detached from each other, and have formed connecting tubes (Figures 2(A2b) and 2(B2b)). Based on published reports [54, 55], such tubes may support exchange of intracellular components between the cancer cells. The activation of FAK and paxillin and their contribution to formation of cellular protrusions were found to be Src-mediated processes. This was indicated by potent inhibition of cell spreading and FAK/paxillin localization at cellular extremities by the specific Src inhibitor PP2 (Figure 3).

**Figure 4:**
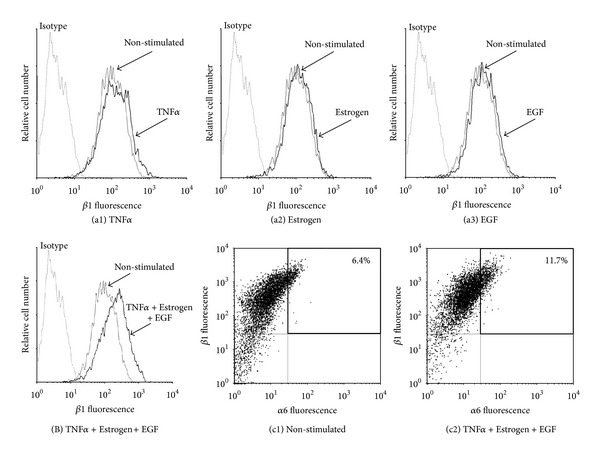
Combined stimulation of breast tumor cells by TNF*α* + Estrogen + EGF leads to increased expression of the *β*1 integrin and to emergence of high-VLA6 cell population (*α*6*β*1^high^). ((a), (B)) Determination of *β*1 expression. Breast tumor cells were stimulated by (a1) TNF*α*, (a2) estrogen, (a3) EGF, or (B) TNF*α* + Estrogen + EGF (concentrations as in Figure 1) for three days. Non-stimulated: cells grown with the diluents of the above factors. The expression of *β*1 on the surface of the cells was determined by FACS analyses, using specific Abs. Isotype: isotype matched Abs used as control in the FACS analyses. In all panels, the results are from a representative experiment of *n* ≥ 3. (c) Coexpression of the *β*1 and *α*6 integrin subunits. (c1) Non-stimulated: cells grown with the diluents of the above factors. (c2) Cells stimulated by TNF*α* + Estrogen + EGF (concentrations as in Figure 1). The expression of *β*1 and of *α*6 on the surface of the cells was determined by FACS analyses using specific Abs, with axes set based on staining with isotype matched control Abs. In all panels, the results are from a representative experiment of *n* ≥ 3.

The powerful spreading induced by TNF*α* + Estrogen + EGF has led us to monitor the expression of the *β*1 integrin, known to be strongly involved in processes of tumor cell adhesion, spreading, and metastasis formation [56–60]. As shown in Figure 4, of the three factors mainly TNF*α* induced detectable upregulation in *β*1 expression although to a very limited extent; however, when TNF*α* activities were joined by estrogen and EGF, the resulting TNF*α* + Estrogen + EGF stimulation has led to much more substantial integrin *β*1 upregulation, in an extent that was stronger than the minimal effects induced by each of the factors alone (Figures 4(a1)–4(a3) versus Figure 4(B)).

The *β*1 integrin has been shown in many studies to stand in the basis of increased adhesion and invasion of tumor cells, including of breast origin [56–60]. Since integrins are acting as *αβ* heterodimers, we searched for the *α* chain counterpart that would accompany the increased expression of *β*1. A thorough search through many different *α* subunits has identified increases in *α*6 in response to TNF*α* + Estrogen + EGF stimulation (Figure 4(c)). Accordingly, the combined stimulation has led to increase in a subpopulation of tumor cells expressing high levels of *α*6*β*1. The *α*6*β*1 heterodimer, otherwise known as VLA6, is a laminin receptor that has been identified in the past as invasion-supporting complex that promotes breast cancer progression [61, 62].

**Figure 5:**
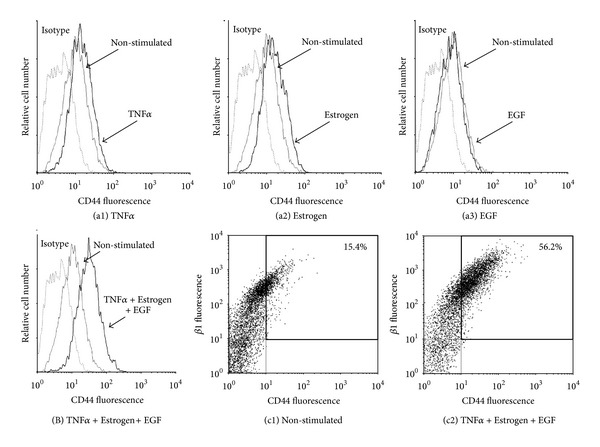
Combined stimulation of breast tumor cells by TNF*α* + Estrogen + EGF leads to potent induction in expression of CD44 and to emergence of a CD44^high^/*β*1^high^ cell population. ((a), (B)) Determination of CD44 expression. Breast tumor cells were stimulated by (a1) TNF*α*, (a2) estrogen, (a3) EGF, or (B) TNF*α* + Estrogen + EGF (concentrations as in Figure 1) for three days. Non-stimulated: cells grown with the diluents of the above factors. The expression of CD44 on the surface of the cells was determined by FACS analyses, using specific Abs to CD44. Isotype: isotype matched Abs used as control in the FACS analyses. In all panels, the results are from a representative experiment of *n* ≥ 3. (c) Coexpression of CD44 and *β*1. (c1) Non-stimulated: cells grown with the diluents of the above factors. (c2) Cells stimulated by TNF*α* + Estrogen + EGF (concentrations as in Figure 1). The expression of CD44 and *β*1 on the surface of the cells was determined by FACS analyses using specific Abs, with axes set based on staining with isotype matched control Abs. In all panels, the results are from a representative experiment of *n* ≥ 3.

In parallel, we found that the combined stimulation by TNF*α* + Estrogen + EGF has induced strong upregulation in another adhesion molecule that is highly implicated in breast metastasis, CD44 (Figure 5) [6, 7, 63, 64]. As previously demonstrated for all other functions, the impact of the combined stimulation on CD44 elevation was definitely more powerful than each of the stimulators—TNF*α*, estrogen, or EGF—alone (Figure 5). Of interest was the fact that due to stimulation by TNF*α* + Estrogen + EGF, over 50% of the tumor cells acquired high expression levels of both *β*1 and CD44 together (Figure 5(c)).

Overall, the above results indicate that of all three factors TNF*α* was the strongest inducer, of spreading and expression of metastasis-related adhesion molecules by the luminal MCF-7 breast tumor cells and that its activities were strongly amplified by the cooperativity with the other two representatives of the tumor microenvironment, estrogen (hormonal) and EGF (growth-supporting).

### 3.2. Combined Stimulation by TNF*α* + Estrogen + EGF Is Advantageous over Each Factor Alone in Inducing EMT in Breast Tumor Cells

**Figure 6:**
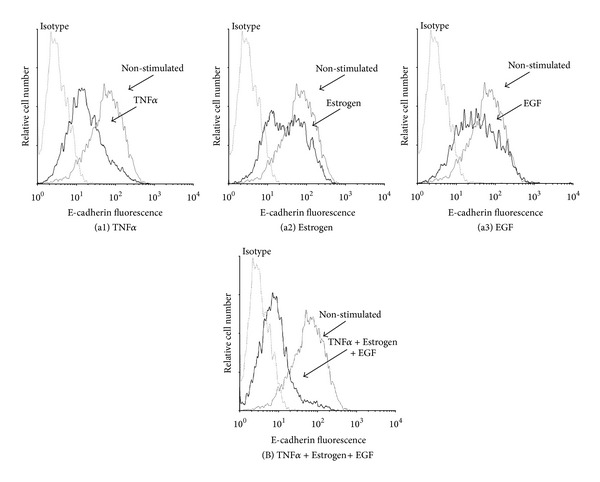
Combined stimulation by TNF*α* + Estrogen + EGF leads to potent downregulation of E-cadherin expression by breast tumor cells. ((a), (B)) Determination of E-cadherin expression. Breast tumor cells were stimulated by (a1) TNF*α*, (a2) estrogen, (a3) EGF, or (B) TNF*α* + Estrogen + EGF (concentrations as in Figure 1) for three days. Non-stimulated: cells grown with the diluents of the above factors. The expression of E-cadherin on the surface of the cells was determined by FACS analyses, using specific Abs to E-cadherin. Isotype: isotype matched Abs, used as control in the FACS analyses. In all panels, the results are from a representative experiment of *n* > 3.

**Figure 7:**
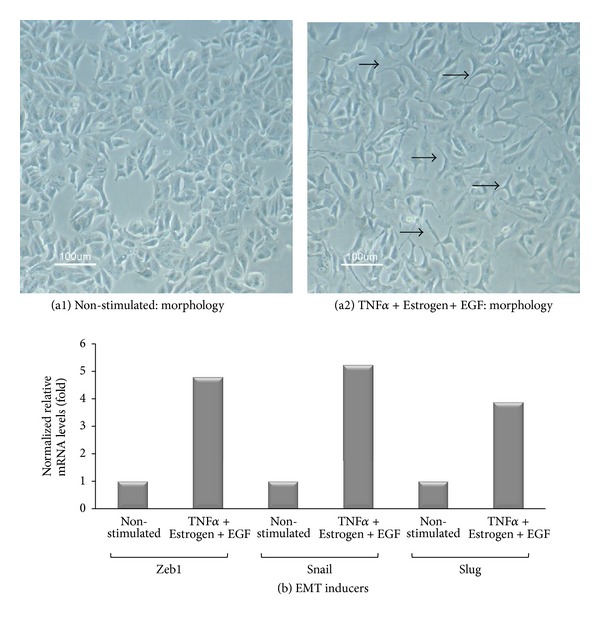
Combined stimulation by TNF*α* + Estrogen + EGF leads to acquisition of mesenchymal phenotype by the tumor cells, and to upregulation of EMT inducers. Breast tumor cells were stimulated by TNF*α* + Estrogen + EGF (concentrations as in Figure 1) for three days. Non-stimulated: cells grown with the diluents of the above factors. (a) Cell morphology was determined by light microscopy at ×20 magnification. (a1) Non-stimulated cells. (a2) Cells stimulated by TNF*α* + Estrogen + EGF. Arrows point to some of the cells that have undergone remodeling in response to TNF*α* + Estrogen + EGF stimulation. In all panels, the results are from a representative experiment of *n* ≥ 3. (b) Expression of the EMT inducers Zeb1, Snail, and Slug in cells stimulated by TNF*α* + Estrogen + EGF and in non-stimulated cells (grown with the diluents of the factors), determined by qPCR analysis. In all panels, the results are from a representative experiment of *n* ≥ 3.

To follow on the above findings, we determined the abilities of TNF*α*, estrogen, and EGF—each alone or together—to induce EMT properties in the tumor cells. In cells undergoing EMT, reduced expression of E-cadherin facilitates detachment of cancer cells from each other [9, 10]. Accordingly, following three days of stimulation by TNF*α*, EGF, and estrogen, each separately, downregulation of cell surface expression of E-cadherin was noted to some extent, with TNF*α* inducing the most prominent effects of all three factors; however, very clearly, the most potent EMT phenotype was obtained upon joint stimulation by all three factors together, given in the form of TNF*α* + Estrogen + EGF (Figure 6). Also, in response to the combined stimulation, the cells have gained typical morphology of cells undergoing EMT, detaching from each other and expressing definite cellular protrusions (Figure 7(a)). Further supporting the ability of TNF*α* + Estrogen + EGF stimulation to induce EMT was the prominent increase in the expression of the known EMT activators Zeb1, Snail, and Slug [65–70] in the tumor cells (Figure 7(b); the EMT regulator twist was down-regulated; data not shown).

### 3.3. Combined Stimulation of Breast Tumor Cells by TNF*α* + Estrogen + EGF Leads to Functional Tumor-Promoting Consequences

**Figure 8:**
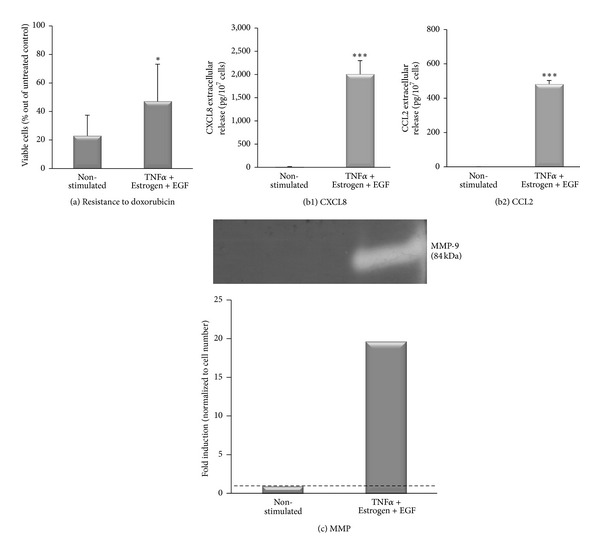
In response to combined stimulation by TNF*α* + Estrogen + EGF, breast tumor cells acquire functional promalignancy properties. Breast tumor cells were stimulated by TNF*α* + Estrogen + EGF (concentrations as in Figure 1) for three days. Non-stimulated: cells grown with the diluents of the above factors. (a) Resistance to doxorubicin. Following their stimulation with the above-mentioned factors, the cells were replated with the aforementioned stimulation in the presence or absence of 1 *μ*M doxorubicin for additional three days. Cell viability was determined by XTT assay. *P < 0.05 for the difference between stimulated and non-stimulated cells. The results are from a representative experiment of *n* ≥ 3. (b) Release of promalignancy factors by the tumor cells, calculated per cell number. The expression of CXCL8 (b1) and CCL2 (b2) was determined in the CM of the cells by ELISA, at the linear range of absorbance. ***P < 0.001 for the differences between stimulated and non-stimulated cells. The results are from a representative experiment of *n* = 3. (c) Release of functional MMPs, determined by zymography assays performed on cell CM. The bar graph shows the quantitative expression of MMPs, calculated per cell number. The results are from a representative experiment of *n* ≥ 3.

Above, we have shown that the combined stimulation by TNF*α* + Estrogen + EGF has strongly induced spreading and EMT properties in luminal breast tumor cells. To follow on the above findings, we determined the impact of the combined stimulation on tumor cell functions that are involved in increased tumor growth and progression. Because of its high clinical relevance to tumor progression, first we asked what is the effect of the combined stimulation on resistance of tumor cells to doxorubicin (adriamycin), which is a chemotherapy commonly used in the treatment of breast cancer patients [71, 72]. When doing this analysis, we were aware of the fact that MCF-7 cells are sensitive to TNF*α* cytotoxicity [73–75], and accordingly our routine procedure of TNF*α* + Estrogen + EGF stimulation for three days has led to death of approximately 40% of the tumor cells (Figure S2). Nevertheless, despite their apparent sensitivity to TNF*α*-induced cytotoxicity, tumor cells that were exposed to the combined stimulation were endowed with higher resistance to doxorubicin (Figure 8(a)). These results indicate that those tumor cells that have survived the TNF*α*-induced cytotoxicity were selected for high resistance to chemotherapy-induced death.

In parallel to the above, we determined the effects of the combined stimulation by TNF*α* + Estrogen + EGF on the ability of the tumor cells to acquire additional promalignancy functions. Doing a “per cell” analysis, we found that the stimulation of the tumor cells by TNF*α* + Estrogen + EGF has given rise to potent elevation in the release of the inflammatory chemokines CXCL8 (Figure 8(b1)) and CCL2 (Figure 8(b2)), which have been well characterized as strong tumor-promoting factors by virtue of their potent angiogenic activities and recruitment of tumor-supporting leukocytes to the tumors [76–81]. In addition, in response to the combined stimulation by TNF*α* + Estrogen + EGF, the tumor cells have acquired the ability to produce high levels of functional matrix metalloproteinase 9 (MMP9; Figure 8(c)), a key enzyme in degradation of the extracellular matrix (ECM) during local invasion end extravasation of the tumor cells [82].

**Figure 9:**
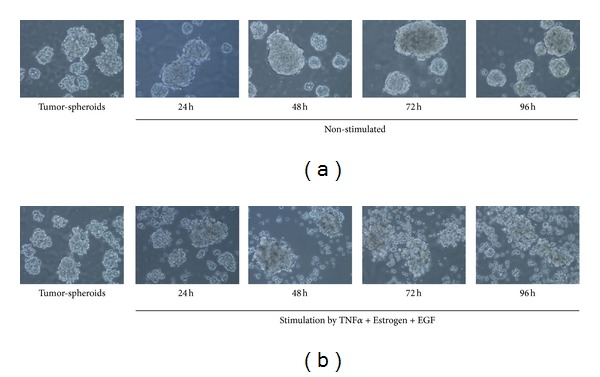
In response to combined stimulation by TNF*α* + Estrogen + EGF, breast tumor cells migrate out of tumor-spheroids. Non-stimulated breast tumor cells were plated in nonadherent conditions, and tumor-spheroids were allowed to form for 72 hr. Then, the cells were either non-stimulated (a), or stimulated by TNF*α* + Estrogen + EGF (concentrations as in Figure 1) (b) for additional 24–96 hr. Non-stimulated cells: cells grown with the diluents of the above factors. Cells were photographed daily using light microscopy at ×10 magnification. Cell viability tests indicated that single cells migrating out of tumor-spheroids formed in the presence of TNF*α* + Estrogen + EGF stimulation were alive. In all panels, the results are from a representative experiment of *n* ≥ 3. The surface expression of E-cadherin by the cells that were included in this analysis is shown in Table 1 and in Figure S3.

Moreover, to follow on the cell-remodeling, EMT, and metastatic/invasive properties acquired by tumor cells that were exposed to the combined stimulation by TNF*α* + Estrogen + EGF, we determined the migratory functions of the cells. We took advantage of the high ability of MCF-7 cells to form tumor-spheroids and analyzed the ability of cancer cells to detach from the spheroids and move away from them. To this end, we have formed tumor-spheroids and then introduced the combined stimulation for additional 24–96 hr. These tests have shown that control, non-stimulated cells, kept the organized spherical structure throughout the 96 hr time course (Figure 9(a)). In contrast, cancer cells that were exposed to the combined stimulation have migrated out of the tumor-spheroids already after 48 hr of stimulation (Figure 9(b)). At the 96 hr time point, extensive outward migration was observed in the TNF*α* + Estrogen + EGF-stimulated cells, and a large proportion of single cells was detected (cell viability tests indicated that these single cells were alive) (Figure 9(b)). Here, it is interesting to note that the single cells that migrated out of tumor-spheroids formed in the presence of TNF*α* + Estrogen + EGF stimulation expressed lower levels of E-cadherin compared to the cells that remained in the spheroids (Table 1 and Figure S3). These results provide a direct connection between the processes of elevated EMT and migratory events that were induced by the combined stimulation of TNF*α* + Estrogen + EGF.

### 3.4. *In Vivo* Animal Studies Indicate That Tumor Cells Stimulated by TNF*α* + Estrogen + EGF Acquire High Metastatic Capacity

The results presented so far in this study indicate that tumor cells exposed to the combined stimulation have acquired properties that may contribute to tumor growth and metastasis. These results have motivated us to determine the effects of the TNF*α* + Estrogen + EGF stimulation on formation of primary tumors at the mammary fat pad and on dissemination of metastasis. To enable detection of the tumor cells in *intact* animals, MCF-7 cells were infected to express the fluorescent protein mCherry. The tumor cells were stimulated for three days by TNF*α* + Estrogen + EGF *in vitro* then washed to remove the stimulators and inoculated to the mammary fat pad of mice. Because MCF-7 cells are sensitive to TNF*α*-induced cytotoxicity (Figure S2) [73–75], following the three days of stimulation by TNF*α* + Estrogen + EGF, we assured that equal numbers of live stimulated and non-stimulated cells were inoculated to the mice. Following tumor cell inoculation, the Maestro device has provided data on the size of primary tumors and appearance of macro-metastases in intact mice, in analyses that were performed at four time points along the course of the experiments (up to 37 days).

**Figure 10:**
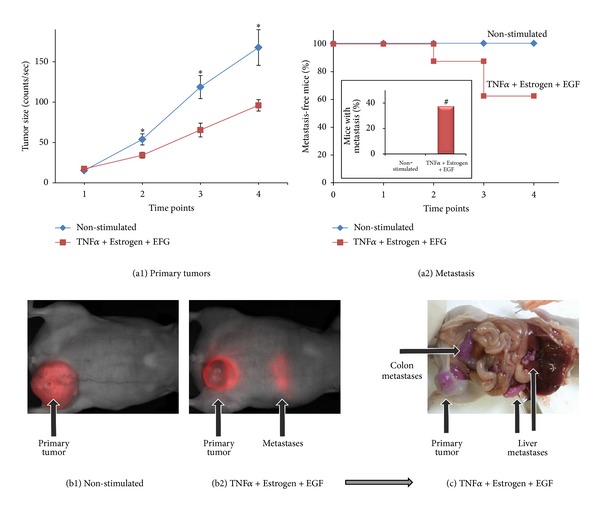
In response to combined stimulation by TNF*α* + Estrogen + EGF, breast tumor cells acquire high metastasizing abilities. mCherry-expressing breast tumor cells were stimulated by TNF*α* + Estrogen + EGF (concentrations as in Figure 1) for three days. Non-stimulated: cells grown with the diluents of the above factors. Following washing, equal numbers of live cells (4 × 10^6^) were inoculated to the mammary fat pad of mice. Using the CRi Maestro intravital imaging system, tumors and metastases were followed in intact mice at four different time points along the experiments, up to 37 days. (a) Followup of primary tumors in the mammary fat pad and formation of macrometastases. (a1) Sizes of tumors at the mammary fat pads are presented as counts/sec of fluorescence emission, divided by 1,000, obtained at each time point by analyses with the CRi Maestro intravital imaging system. *P < 0.05 for differences between the two groups of mice. The figure sums up the results obtained in two experimental repeats showing similar results, with a total *n* = 6 mice in the control group *n* = 8 mice in the group of mice inoculated with cells stimulated with TNF*α* + Estrogen + EGF. (a2) Kaplan-Meier analyses of metastasis-free mice, showing incidence of macrometastases detected by the Maestro device in intact animals in four time points along the experiments, up to 37 days. The figure sums up the results obtained in two experimental repeats showing similar results, with a total of *n* = 6 mice in the control group and *n* = 8 mice in the group of mice inoculated with cells stimulated with TNF*α* + Estrogen + EGF. *Inset*: the incidence of mice with macrometastases at the end-point of the experiments, determined in intact mice by the Maestro device (38% in the TNF*α* + Estrogen + EGF-stimulated tumor cells versus 0% in the control group, in two independent experiments providing similar results). ^*#*^ *Macrometastases were also observed in 2/3 mice in another experiment of TNF*α* + Estrogen + EGF cells* (in which control mice were not included). (b) Representative pictures obtained by the Maestro device in intact mice, showing tumor cells (red, mCherry) in both groups of mice. Non-stimulated tumor cells (b1) developed bigger tumors than tumor cells stimulated with TNF*α* + Estrogen + EGF (b2); however, macrometastases were detected only in the group of mice administered with tumor cells stimulated by TNF*α* + Estrogen + EGF (the image was obtained following prolonged excitation of mCherry in the CRi Maestro, in order to visualize the metastases). (c) A representative picture of the macrometastases that have developed in mice inoculated with tumor cells stimulated by TNF*α* + Estrogen + EGF, at the end of the experiment. The image shows the same mouse demonstrated in part (b2). Because of the expression of mCherry, the tumor cells carried a purple color. In this representative mouse, metastases were detected in the liver, colon, and above the kidney.

The findings of Figure 10(a1) show that cells exposed to the combined stimulation of TNF*α* + Estrogen + EGF have given rise to smaller tumors than control non-stimulated cells, due to possible reasons described further on (Section 4). However, a totally different picture was revealed when metastasis formation was addressed. Taking into account the fact that MCF-7 cells are well-characterized as nonmetastatic cells [45, 83], it was exciting to see that the combined stimulation by TNF*α* + Estrogen + EGF for three days in culture has given rise to cells with high metastasizing ability *in vivo*. As expected, the control cells did not form macro-metastases at all, but in contrast the tumor cells that have been exposed to TNF*α* + Estrogen + EGF stimulation have given rise to macro-metastases in 38% of the animals (Figures 10(a2), 10(b), and 10(c)), as determined in 2 independent experimental repeats showing similar results. Macro-metastases were also detected in 2/3 mice in another experiment in which non-stimulated cells were not included. The macro-metastases formed by TNF*α* + Estrogen + EGF-stimulated cells were detected in the liver, colon, and abdomen (Figure 10(d) shows metastases in the liver and in the colon).

Actually, the impact of the combined stimulation on the metastatic potential of the MCF-7 was dramatic: the tumor cells were exposed to this stimulus for only three days in culture, and based on our *in vitro* results only a subpopulation (based on Figure 5, up to ~50% of the cells) has gained tumor and metastasizing abilities in culture (Figures 4–6). Also, in other *in vivo* studies that we have performed with oncogene-expressing MCF-7 cells that were stimulated by TNF*α* (in which mice were also injected twice-weekly with CM of such cells) suggest that the metastatic load of cells stimulated by TNF*α* + Estrogen + EGF is higher than the one induced by TNF*α* (data not shown). Taken together, the influence of the TNF*α* + Estrogen + EGF stimulation on the metastasizing capabilities of these cells *in vivo* is of major importance and of high clinical relevance.

## 4. Discussion

In this study, we demonstrated that the combination between promalignancy factors has a dramatic impact on the ability of luminal tumor cells to acquire metastasis-related properties and to disseminate to remote organs. When used singly, TNF*α* was more effective than the other two representatives of the tumor microenvironment—estrogen and EGF—and its activities were potently increased by cooperating with these two factors. Thus, it was the joint activities of all three arms together—inflammatory, hormonal, and growth-supporting—that led in a prominent efficacy to the devastating processes of tumor cell spreading, EMT, and metastasis.

Our findings have shown that as a result of the combined stimulation by TNF*α* + Estrogen + EGF, luminal breast tumor cells have gained an extensive spreading phenotype in which Src activation has given rise to tumor cell spreading and to localization of FAK and paxillin in tumor cell protrusions. In parallel, the cancer cells have detached from each other and underwent the metastasis-relevant process of EMT and migration. As a result of TNF*α* + Estrogen + EGF stimulation, new cell subtypes have dominated the tumor cell population, expressing high levels of VLA6 and of the metastasis-related adhesion molecules CD44 and *β*1, accompanied by high levels of CXCL8, CCL2, and MMPs that were released by the cells. Based on published findings [84–87], the elevation in *β*1, CD44, and CXCL8 may very much stand in the basis of the high resistance to doxorubicin gained by the TNF*α* + Estrogen + EGF-stimulated cells.

The above characteristics that were gained by the tumor cells following exposure to TNF*α* + Estrogen + EGF have led to an intriguing *in vivo* phenotype, in which the stimulated cells have produced smaller local tumors but expressed very high metastatic phenotype compared to control non-stimulated cells. Based on the *in vitro* results described previously, two nonexclusive mechanisms could lead to such results: (1) out of the three stimulators of the tumor cells, TNF*α* is the only one that is cytotoxic while estrogen and EGF are known to stimulate tumor cell growth. The tumor cells used in this study (MCF-7 cells) are known to be sensitive to TNF*α*-induced cytotoxicity [73–75]; accordingly, approximately 40% of the tumor cells were killed *in vitro* by their exposure to the combined stimulation of TNF*α* + Estrogen + EGF (Figure S2). Although after this stimulation only live cells were injected (in equal numbers to control cells) to the mice and the stimulus was removed beforehand, it is possible that some of the tumor cells were destined to die later on, after they have been introduced into the mouse. These cytotoxic effects of TNF*α* may have given rise to reduced growth of primary tumors. (2) the high spreading, EMT, and migration phenotypes endowed on the cancer cells by TNF*α* + Estrogen + EGF stimulation may have led to migration of tumor cells out of the primary focus soon after their inoculation to the mammary fat pad (as has been illustrated *in vitro* in Figure 9); thus, the cell inoculum from which the tumor developed was smaller after stimulation and gave rise to a smaller primary tumor than control non-stimulated cells. Such a mechanism is in good agreement with the high metastatic yield of the TNF*α* + Estrogen + EGF-stimulated cells (Figure 10). Obviously, such mechanisms suggest that it would be interesting to determine the EMT properties in primary tumors established by non-stimulated control cells compared to cells stimulated by TNF*α* + Estrogen + EGF.

Overall, our results suggest that some of the cancer cells that were stimulated by TNF*α* + Estrogen + EGF partly succumbed to the cytotoxic effects of TNF*α* and others migrated out of the initial tumor inoculum, giving rise to smaller primary tumors than those generated by control cells. But at the same time, these cells have gained many metastasis-promoting properties and became aggressive *in vivo*. Therefore, the small tumor growth endowed following TNF*α* + Estrogen + EGF stimulation provided a false benefit, because it has led to selection of cells expressing a higher metastasizing potential. Here, it is important to note that in the heterogeneous population of tumor cells, only some have acquired the high “spreading-EMT-metastasis"-related functions, and this can explain why metastases were not formed in all mice. Nevertheless, we would like to emphasize that the acquisition of a metastatic ability by MCF-7 cells is by itself extremely unique and important, even if not observed in all mice. MCF-7 cells are considered completely nonmetastatic, and even following over-expression of powerful proto-oncogenes such as H-Ras, they did not acquire the ability to form metastases *in vivo*, despite increased invasiveness *in vitro* [83].

Therefore, our findings indicate that under certain conditions—endowed by combined stimulation by three arms of the tumor microenvironment—MCF-7 cells became metastatic. In our *in vitro* analyses, TNF*α* was the most effective of all three elements, but its activities were potentiated by estrogen and EGF. Based on these studies, we propose that TNF*α* is the factor that dominated the high protumoral phenotypes and responses, leading to its most extreme impact on tumor cell spreading to remote organs.

Overall, while TNF*α* had the ability to exert cytotoxic effects that may reduce tumor growth, it cooperated with the two other arms of the tumor microenvironment and eventually turned into a metastasis-promoting entity. Here, it is important to note that all three factors—TNF*α*, estrogen, and EGF—are often expressed in luminal breast tumors in breast cancer patients. Past findings from our laboratory indicated that TNF*α* is expressed in approximately 90% of patients with recurrent disease, and many of these patients also express ER, and are therefore estrogen-responsive [22]. Other studies denoted that about 70% of breast tumors express the ligand EGF [88]. Taken together, these observations suggest that a relatively high subpopulation of luminal breast cancer patients may experience coexposure to TNF*α* + Estrogen + EGF and may thus acquire increased metastatic rate. Moreover, based on our results with doxorubicin resistance, the joint powers of all three factors together may further increase the resistance to chemotherapy in breast cancer patients, demonstrating another level at which the combined exposure to TNF*α* + Estrogen + EGF may be devastating to the patients.

## 5. Conclusions

The findings presented in this study have very high clinical relevance. Until a decade ago, many researchers suggested introducing TNF*α* as a therapeutic agent in cancer because of its cytotoxic activities. However, an increasing body of evidence puts TNF*α* on the stake as a key tumor-promoting factor that has harmful impacts on the malignancy cascade. Our findings pinpoint the devastating TNF*α* activities to be the life-threatening stage of metastasis formation, and these findings have a profound importance for breast cancer therapy. TNF*α* inhibitors, such as infliximab and etanercept have been FDA-approved and are being successfully used in the clinic for treatment of several autoimmune disorders [89–92]. Therefore, we suggest considering the addition of these established TNF*α* inhibitors to the treatment protocols of luminal breast cancer patients.

Specifically, we suggest taking the results of this study one step further, towards personalized cancer therapy. Knowing that antihormone therapies and inhibitors of EGFR/HER2 are already used for therapy of breast cancer [93, 94], we propose that patients diagnosed with high TNF*α*, estrogen, and EGF levels would benefit from targeting all three arms simultaneously and that clinicians should consider the possibility of treating such patients with a cocktail of all three modalities: TNF*α* inhibitors + antihormonal therapies + inhibitors of EGFR/HER2.

Obviously, extensive research is needed in order to assess the impact of TNF*α* inhibitors on breast tumor cells both *in vitro* and *in vivo*, and to design the proper clinical administration mode. However, we believe that the paradigm shift presented in this study on the roles of TNF*α* in metastasis may have a strong impact on therapeutic choices in the future. The feasibility of blocking several arms of the tumor microenvironment together should not be ignored, and reducing the cancer-related inflammation might also attenuate the tumor-promoting effects imposed by the other arms of the tumor microenvironment and thus inhibit tumor cells migration and invasion and their devastating outcome, metastasis formation.

## Supplementary Material

Supplementary Figure 1: Combined stimulation by TNF**α**+Estrogen+EGF is more effective than Estrogen+TNF**α** or Estrogen+EGF in inducing extensive morphological changes and spreading in breast tumor cells.
Supplementary Figure 2: Combined stimulation by TNF**α**+Estrogen+EGF leads to reduced viability of breast tumor cells in culture.
Supplementary Figure 3: Breast tumor cells that migrated out of tumor-spheroids following TNF**α**+Estrogen+EGF stimulation, express reduced levels of E-cadherin.

## Figures and Tables

**Table 1:** Breast tumor cells that migrated out of tumor-spheroids following TNF*α* + Estrogen + EGF stimulation express reduced levels of E-cadherin.

E-cadherin expression	Cells dissociated mechanically from tumor-spheroids formed by non-stimulated cells	Cells dissociated mechanically from tumor-spheroids formed in the presence of TNF*α* + Estrogen + EGF stimulation	Single cells that have detached spontaneously from tumor-spheroids formed in the presence of TNF*α* + Estrogen + EGF stimulation
Mean fluorescence (MFI)	15.8	15.4	11.5
% Positive cells	18.0	20.9	5.7
Score (MFI × %)	284.4	321.9	65.5
Normalized values	1.00	1.13	0.23

The table summarizes the analyses performed for E-cadherin expression in tumor-spheroid assays. The formation of tumor-spheroids and the ability of TNF*α* + Estrogen + EGF-stimulated cells to migrate out of the tumor-spheroids were shown in Figure 9. Then, tumor-spheroids and single cells were separated by a 40 *μ*m nylon mesh. Cells from tumor-spheroids formed in the absence or in the presence of TNF*α* + Estrogen + EGF stimulation were dissociated mechanically by trypsinization and were stained for E-cadherin in comparison to cells that have detached spontaneously from the tumor-spheroids formed in the presence of TNF*α* + Estrogen + EGF stimulation. The three cell types were analyzed for surface expression of E-cadherin by flow cytometry analyses. The results are from a representative experiment of *n* ≥ 3.

## Abbreviations

Abs: Antibodies
CM: Conditioned medium
DMSO: Dimethyl sulfoxide
EGF: Epidermal growth factor
EGFR: Epidermal growth factor receptor
EMT: Epithelial-to-mesenchymal transition
ER: Estrogen receptors
FACS: Fluorescence-activated cell sorting (flow cytometry)
FAK: Focal adhesion kinase
HRP: Horseradish peroxidase
MMPs: Matrix metalloproteinases
PR: Progesterone receptors
qRT-PCR: Quantitative real-time polymerase chain reaction
TNF*α*: Tumor necrosis factor *α*
PFA: Paraformaldehyde
DAPI: 4′,6-Diamidino-2-phenylindole.
