# Supplementary material for: Progression of Luminal Breast Tumors Is Promoted by Ménage à Trois between the Inflammatory Cytokine TNFα and the Hormonal and Growth-Supporting Arms of the Tumor Microenvironment
Source: Mediators Inflamm. 2013 Dec 4;2013:720536. doi: 10.1155/2013/720536 (PMC3867893; doi:10.1155/2013/720536)
Supplement: Supplementary file 1 — Supplementary Figure 1: Combined stimulation by TNFα+Estrogen+EGF is more effective than Estrogen+TNFα or Estrogen+EGF in inducing extensive morphological changes and spreading in breast tumor cells. Supplementary Figure 2: Combined stimulation by TNFα+Estrogen+EGF leads to reduced viability of breast tumor cells in culture. Supplementary Figure 3: Breast tumor cells that migrated out of tumor-spheroids following TNFα+Estrogen+EGF stimulation, express reduced levels of E-cadherin. [file 720536.f1.pdf]

Fig. S1

A1. Non-stimulated

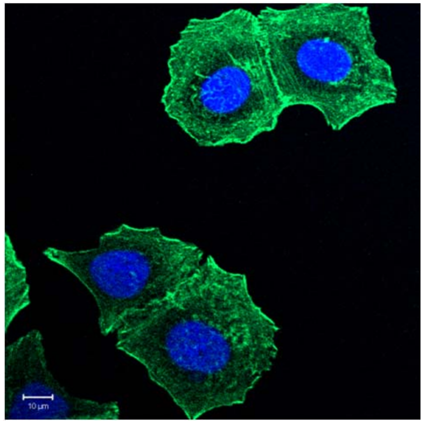

A2a. Estrogen+TNFα

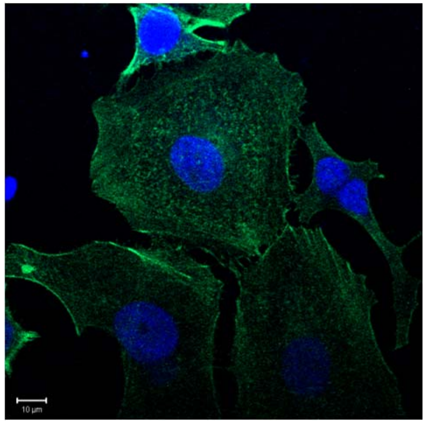

A2b. Estrogen+EGF

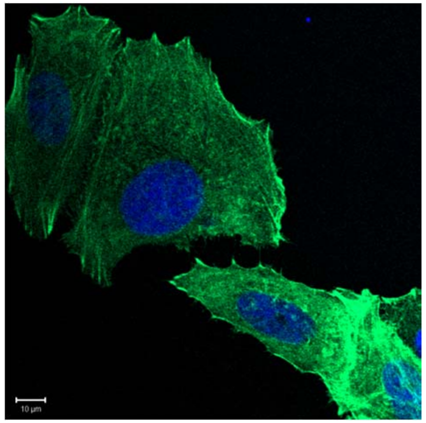

B. TNFα+Estrogen+EGF

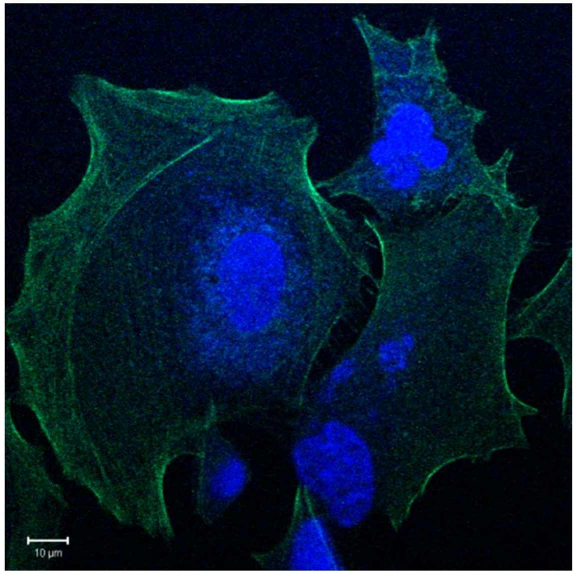

Fig. S2

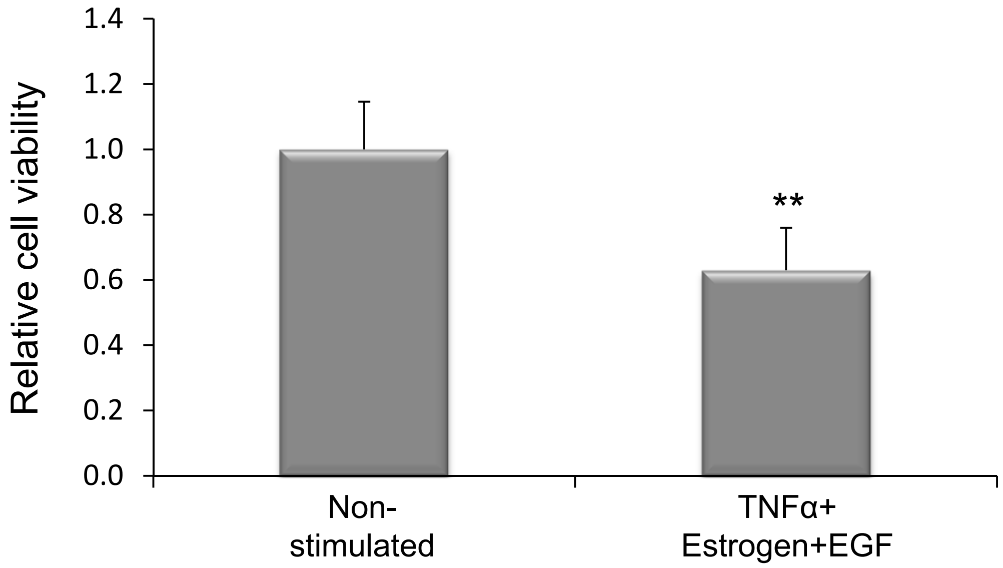

Relative cell viability
1.4
1.2
1.0
0.8
0.6
0.4
0.2
0.0
**
Non-stimulated
TNFα+ Estrogen+EGF

Fig. S3

A. Control vs. TNFα+Estrogen+EGF: Cells from tumor-spheroids

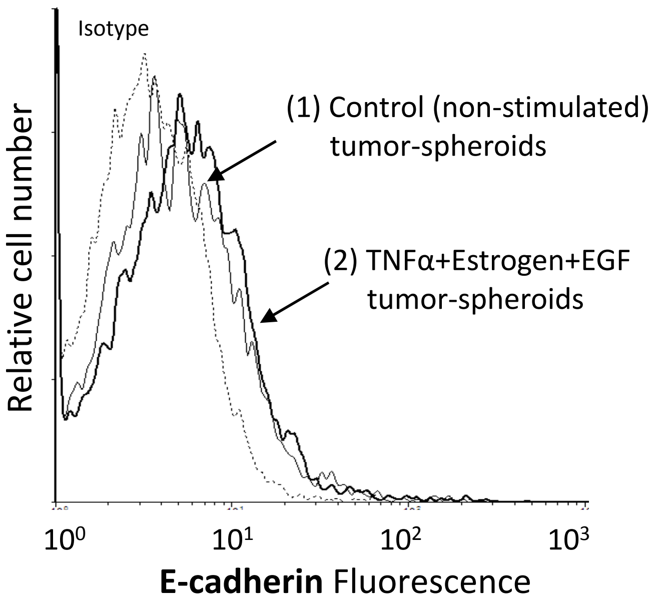

B. TNFα+Estrogen+EGF : Cells from tumor-spheroids vs. single cells

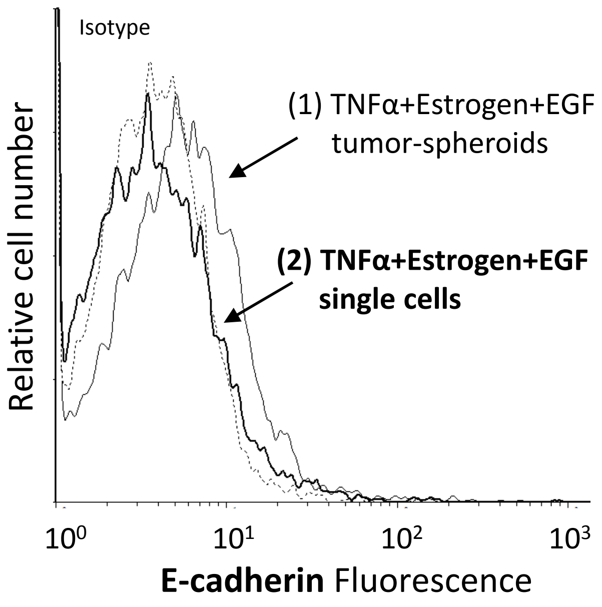

Legends to supplementary figures

Figure S1

Combined stimulation by TNFα+Estrogen+EGF is more effective than Estrogen+TNFα or Estrogen+EGF in inducing extensive morphological changes and spreading in breast tumor cells

Breast tumor cells were either (A1) not stimulated (=cells grown in the presence of diluents) or stimulated by (A2a) Estrogen+TNFα (10^{-8} M and 50 ng/ml, respectively), (A2b) Estrogen+EGF (10^{-8} M and 30 ng/ml, respectively) or (B) TNFα+Estrogen+EGF (concentrations as above) for three days. The stimulatory conditions were selected following titration and kinetics analyses (Data not shown). Actin filaments were detected by phalloidin staining (green) and cell nuclei by DAPI staining (blue). The cells were analyzed by confocal microscopy. In all panels, the results are from a representative experiment of $n \geq 3$.

Figure S2

Combined stimulation by TNFα+Estrogen+EGF leads to reduced viability of breast tumor cells in culture

Breast tumor cells were stimulated by TNFα+Estrogen+EGF (concentrations as in Fig. 1) for three days. Non-stimulated = Cells were grown with the diluents of the above factors. Cells were removed from growth plates and live cells were counted by trypan blue. Cell numbers in the control group of non-stimulated cells were given the value of 1. **$p<0.01$ for differences between stimulated and non-stimulated cells. The figure presents the average ± SD values obtained in n=4 experiments.

Figure S3

Breast tumor cells that migrated out of tumor-spheroids following TNFα+Estrogen+EGF stimulation, express reduced levels of E-cadherin

Breast tumor cells were stimulated by TNFα+Estrogen+EGF (concentrations as in Fig. 1) for three days. Non-stimulated = Cells grown with the diluents of the above factors. Tumor-spheroids were produced, as shown in Figure 9, and later separated to tumor-spheroids and single cells by a 40μM nylon mesh. Cells from tumor-spheroids were dissociated mechanically by trypsinization, and were stained for E-cadherin in comparison to cells that have detached spontaneously from the tumor-spheroids. **(A)** Determination of E-cadherin expression in (1) Tumor cells that were dissociated mechanically from tumor-spheroids formed by control non-stimulated cells, and (2) Tumor cells that were dissociated mechanically from tumor-spheroids formed by TNFα+Estrogen+EGF-stimulated cells. **(B)** Determination of E-cadherin expression in (1) Tumor cells that were dissociated mechanically from spheroids formed by TNFα+Estrogen+EGF-stimulated cells, and (2) Tumor cells that migrated spontaneously out of the tumor-spheroids formed by TNFα+Estrogen+EGF-stimulated cells. The cells that have migrated out of the tumor-spheroids are referred to as "single cells". In each panel, the figure shows a representative experiment in which the surface expression of E-cadherin was determined by FACS analysis, using specific Abs to E-cadherin. Isotype = Isotype matched control Abs, used as control in the FACS analyses. The results are from a representative experiment of n≥3.
